# *In vitro* fermentation and production of methane and carbon dioxide from rations containing *Moringa oleifera* leave silage as a replacement of soybean meal: *in vitro* assessment

**DOI:** 10.1007/s11356-022-20622-2

**Published:** 2022-05-16

**Authors:** Tarek A. Morsy, Gouda A. Gouda, Ahmed E. Kholif

**Affiliations:** Dairy Science Department, National Research Centre, 33 Bohouth St. Dokki, Giza, Egypt

**Keywords:** Degradability, Ensiling, Foliage feeds, Greenhouse gases, *In vitro* fermentation, Unconventional protein feeds

## Abstract

Plant leaf meal of some forage trees such *as Moringa oleifera* has attracted an increasing interest as a good and cheap source of protein. The present *in vitro* experiment employed the *in vitro* wireless gas production (GP) technique to evaluate the inclusion of *M. oleifera* leaves ensiled for 45 days as a replacement for soybean meal in rations. A control basal ration was formulated to contain 17.5% soybean meal as a source of protein. Soybean meal in the control ration was replaced with silage (MOS) at increasing levels of 0 to 100%. Replacing soybean meal with MOS gradually increased (*P* < 0.001) GP kinetics (asymptotic GP, rate of GP, and lag time of GP). However, soybean meal replacement decreased (*P* < 0.001) asymptotic methane (CH_4_) and carbon dioxide (CO_2_) productions, and rate of CH_4_ production and increased the lag time of CH_4_ and CO_2_ production. Gradual increases (*P* < 0.001) in the digestibility of dry matter, neutral detergent fiber and acid detergent fiber, ruminal bacteria count, fermentation pH, and the concentrations of ruminal total volatile fatty acids, acetate, and propionate were observed with rations containing MOS. Decreases in the digestibility of crude protein, ruminal protozoal count, and the concentrations of ruminal ammonia-N were observed with MOS rations. It is concluded soybean meal can be completely replaced by MOS with desirable effects on ruminal fermentation.

## Introduction

Protein feeds are the most important components of animal’s ration. Due to their scarcity and high prices, it is necessary to explore and evaluate unconventional plants, rich in protein, as suitable and viable alternatives to conventional animal protein feeds (Abarghuei and Salem 2021; Özelçam et al. 2021). Protein-rich plant leaf meals, with a good amino acid profile and low prices, can be used as suitable alternatives (Kholif et al. 2016). In their review, Kronqvist et al. (2021) noted that feeding foliage to goats increased feed intake and average daily weight gain compared with grass-based diets. Additionally, they observed a decreased neutral detergent fiber (NDF) and higher rotein (CP) in the foliage than in the grass. Özelçam et al. (2021) evaluated the nutritive value of ensiled or dried *Paulownia* spp. leaves and observed a considerable *in vitro* organic matter digestibility, metabolizable energy value, and rumen fermentation characteristics, making them good feeds for ruminants. *Moringa oleifera* is an another excellent example of protein leaf meal and secondary metabolites that showed good results as an alternative for conventional protein feeds in ruminants (Kholif et al. 2016).

*M. oleifera*, an indigenous tree native to the Himalaya, has been widely distributed almost worldwide. It can be grown successfully in a variety of conditions such as hot, humid, dry, and subtropical tropics, with multiple time harvests. The silage of *M. oleifera* leaves contains about 30% CP (dry matter (DM) basis) of the whole plant, with a high concentration of bypass protein and an adequate amino acid profile (Ebeid et al. 2020a). *In vitro* evaluation (Ebeid et al. 2020a), and experiments on lactating goats (Kholif et al. 2018), sheep (Kewan et al. 2019), and calves (Abdel-Raheem and Hassan 2021), promising results, including feed utilization, milk production and composition, and milk fatty acids profile, were reported. Kholif et al. (2016) evaluated fresh or ensiled leaves of *M. oleifera* as a replacement of sesame meal at different levels in the diet of lactating goats and observed improved feed digestion, ruminal fermentation, and milk production and composition. Using* in sacco* fermentation, Ebeid et al. (2020a) evaluated *M. oleifera* leaves and seeds and observed that leaves showed better nutritive value compared to seeds*.* They observed that *M. oleifera* leaves had a high DM disappearance and effective degradability. Additionally, Ebeid et al. (2020b) observed that *M. oleifera* increased microbial protein and propionate concentration and decreased ruminal protozoal and methanogen counts.

It is well documented that secondary metabolites in some plant species can mitigate methane (CH_4_) production from ruminal fermentation (Parra-Garcia et al. 2019; Kholif and Olafadehan 2021). *M. oleifera* is a plant rich in secondary metabolites including tannins, saponins, and many other phenolic compounds (Kholif et al. 2016). Such compounds have the ability to suppress methanogens such as *Methanobrevibacter* spp., *Methanomicrobium* spp., *Methanobacterium* spp., *Methanosarcina* spp., and methanogenic archaea (Kholif and Olafadehan 2021). Saponins reduce CH_4_ production via inhibition of ruminal protozoa (Ebeid et al. 2020b). Tannins had the potential to reduce CH_4_ production by about 50% (Goel and Makkar 2012) due to their antimicrobial properties, which inhibit some ruminal CH_4_-producing bacteria and protozoa by binding dietary proteins and microbial cell enzymes (Bodas et al. 2012).

The aim of this experiment was to evaluate the potential use of green, cheap, and readily available foliage (i.e., *M. oleifera*) as a replacement of soybean meal, the costliest feedstuff in livestock ration formulation, to increase farmers’ gain and reduce environmental pollution. Therefore, this experiment evaluated the effects of replacing soybean meal with different levels (0 to 100%) of *M. oleifera* silage (MOS) on *in vitro* gas production (GP), biogases production, nutrient degradability, and ruminal fermentation. We hypothesized that the relatively high CP content of *M. oleifera* leave silage (24.2% DM basis), good amino acids profile, and moderate secondary metabolites would improve ruminal fermentation and decrease biogas production when used as a replacement for soybean meal. Additionally, it was hypothesized that the low CP degradability of MOS (high bypass protein) would match the high CP concentration in soybean meal (high CP degradability and low bypass protein).

## Materials and methods

### *M. oleifera* cultivation

*M. oleifera* seeds were planted at a density of 100,000–150,000 seeds per ha (Kholif et al. 2016). The field was irrigated (900 m^3^ water/ha) twice a week without fertilizer. The first cutting was carried out after 65 days at about 65–70 cm height. Due to the high moisture content, the first cut was not used. The second cut of *M. oleifera* was taken after 45 days and used for the *in vitro* evaluation. After cutting, leaves and young twigs were left in the field for an hour then cut into small pieces, and molasses were added at 5% fresh weight. The materials were then manually packed into polythene bags (40 × 70 cm) and compressed to create anaerobic conditions. The bags were hermetically sealed and stored in dry conditions for 45 days. A small amount of the ensiled material was dried and kept for the *in vitro* evaluation and chemical analysis.

### Experimental rations

Control basal ration, containing (per kg DM) berseem hay and a concentrate feed mixture at 1:1 DM basis, was formulated. The concentrate feed mixture contained (DM basis) 400 g corn grains, 200 g wheat bran, 350 g of soybean meal, and 50 g vitamins/minerals mixture. Ten rations, in which soybean meal was replaced with dried MOS at 10, 20, 30, 40, 50, 60, 70, 80, 90, and 100%, were formulated and used for the *in vitro* incubation. Feed samples (ingredients and rations) were dried at 60 °C in a forced-air oven for 48 h then mixed and ground to pass a 1-mm screen in a mill. The ground samples were stored for chemical analyses. The ingredient and chemical composition of the formulated rations used as substrates is presented in Table 1.

**Table 1:** Ingredients and chemical composition of ingredients and rations^1^ (g/kg DM)

	Berseem hay	Soybean meal	MOS^2^	Wheat bran	Yellow corn	DM	OM	CP	EE	NSC	NDF	ADF
Ingredient												
Berseem hay						860	858	193	32	217	416	302
Soybean meal						890	935	438	30	278	189	120
MOS^2^						869	899	242	40	299	318	291
Wheat bran						871	852	130	130	131	462	131
Yellow corn						866	890	91	91	494	214	89
Replacement^1^												
0% (control)	500	175	0	100	200	846	856	204	52.2	269	330	203
10%	500	157.5	17.5	100	200	846	855	201	52.4	269	332	206
20%	500	140	35	100	200	845	855	198	52.6	270	335	209
30%	500	122.5	52.5	100	200	845	854	194	52.8	270	337	212
40%	500	105	70	100	200	845	853	191	52.9	270	339	215
50%	500	87.5	87.5	100	200	844	853	187	53.1	271	342	218
60%	500	70	105	100	200	844	852	184	53.3	271	344	221
70%	500	52.5	122.5	100	200	844	851	180	53.5	272	346	224
80%	500	35	140	100	200	843	851	177	53.6	272	348	227
90%	500	17.5	157.5	100	200	843	850	174	53.8	272	351	230
100%	500	0	175	100	200	842	850	170	54.0	273	353	232

*ADF*, acid detergent fiber; *CP*, crude protein; *DM*, dry matter; *EE*, ether extract; *MOS*, *M. oleifera* silage; *NDF*, neutral detergent fiber; *NSC*, non-structural carbohydrates; *OM*, organic matter

^1^* M. oleifera* silage replaced soybean meal at 0 to 100%, DM basis.

^2^*M. oleifera* silage measurements: pH = 4.2, ammonia-N = 51 g/kg of total N, volatile fatty acids = 88 g/kg DM, aflatoxin F_1_ = 1.1 µg/kg of DM, total phenolics = 42 g/kg DM, tannins = 19 g/kg DM, saponins = 64 g/kg DM

### Feed analysis

Following the official methods of AOAC (2005), samples of MOS, feed ingredients, and rations were analyzed for N content using Kjeldahl method. Ether extract (EE) content was measured using petroleum ether in a Soxhlet extractor. Ash content was measured after burning the samples in a muffle furnace at 550 °C. Concentrations of non-structural carbohydrate (NSC = 1000–NDF–CP–EE–ash) and organic matter (OM = 1000–ash) were calculated. NDF content was determined according to Van Soest et al. (1991) with the use of sodium sulfite and alpha amylase. ADF concentration was analyzed and expressed exclusive of residual ash as described in the AOAC (2005) official method. 

Concentrations of tannin (Makkar 2003), total phenolic (Meier et al. 1988), and saponins (Farajzadeh et al. 2020) were determined in MOS. Additionally, the quality of MOS was assessed by measuring pH, volatile fatty acids (VFA), and N-ammonia as detailed by Kholif et al. (2022). Aflatoxin (AF_1_) concentration was measured in MOS using a fluorometer (Series-4, VICAM, USA), based on the methods described by AOAC (2005).

### *In vitro* fermentation and biodegradation

As previously detailed by Ebeid et al. (2022), the *in vitro* ruminal fermentation was performed using 250-mL bottles (ANKOM^RF^ Gas Production System) fitted with an automatic wireless GP module (Ankom Technology, Macedon, NY, USA) and pressure sensors. An incubation medium (buffer, macromineral, micromineral, and resarzurin solutions) was prepared (Goering and Van Soest 1970) in a volumetric flask at 39 °C. To remove O_2_ from the buffer solution, a reduction agent (sodium sulfide solution) was added (2 mL) to the buffer shortly before rumen fluid addition. In each incubation module (250 mL bottle), 20 mL of ruminal inoculum was mixed with 80 ml of buffer, while allowing a head space of 150 mL.

Rumen inoculum was collected from the rumen of three slaughtered Barki rams (51.7 kg body weight) in a local slaughterhouse. The rams were *ad libitum* fed a diet containing concentrates, berseem hay, and rice straw at 500:400:100 (DM basis), with free access to water. The rumen contents were individually collected from each ram in a thermos and maintained at 39 °C until transported to the laboratory where a little carbon dioxide (CO_2_) was added. Upon arrival to laboratory, ruminal fluid was filtered through four-layered cheesecloth and then the particulate materials were squeezed to obtain microbes attached to feed particles. Ruminal fluids from the rams were mixed before use.

The control and formulated rations were tested in three bottles (analytical replicates) and two incubation runs in 2 successive weeks with 2 bottles containing inoculum and buffer but no feed (blanks) (11 treatments × 3 replicates × 2 incubation runs + 2 blank bottles). A 1 g ± 10 mg sample for each ration was weighed into filter bags (ANKOM F57; Ankom Technology, Macedon, NY, USA) and the bags were put into 250-mL bottles.

Accumulated gas pressure converted to volume (ml) at standard pressure and temperature was measured. The average GP in the empty bottles (empty corrected GP) was subtracted to obtain the net GP after 2, 4, 6, 8, 10, 12, 16, 20, 24, 36, 48, 72, and 96 h of starting the incubation. At each incubation time, 5 mL of gas was taken from the sampling hole of each module and injected into a Gas-Pro detector (CROWCON Model Tetra3 Gas Analyzer, Abingdon, UK) to measure CH_4_ and CO_2_ concentrations in the total gas (Kholif et al. 2022).

After 96 h of incubation, bottles were swirled in ice for 5 min to terminate the incubation, and the pH was measured immediately. The filter bags were removed from the bottles and dried in a forced air oven at 55 °C for 48 h. The degradations of DM, NDF, and ADF were calculated by difference between the initial (substrates) and final (residues) weights of the dried substrate DM or NDF or ADF, respectively.

At the end of incubation, 5 mL of the fluid samples was collected from each bottle in glass tubes for measuring ammonia-N and total and individual VFA concentrations as detailed by Kholif et al. (2022). Individual VFA were measured using a chromatography after processing 1.6 mL of strained rumen fluid with 0.4 mL of a solution containing 250 g of metaphosphoric acid as described previously using a gas chromatograph (GC, Thermo fisher scientific, Inc., TRACE1300, Rodano, Milan, Italy). The GC was fitted with an AS3800 autosampler and equipped with a capillary column HP-FFAP (19091F-112; 0.320 mm o.d., 0.50 μm i.d., and 25 m length; J & W Agilent Technologies Inc., Palo Alto, CA, USA). A mixture of known concentrations of individual VFA was used as an external standard (Sigma Chemie GmbH, Steinheim, Germany) to calibrate the integrator (Kholif et al. 2022).

Samples of fermented fluid (4 mL) were individually mixed with 4 mL of methyl green-formalin-saline solution and stored in a refrigerator at 4 °C until analysis of bacterial and protozoal count following the procedure described by Dehority (1993). Total bacteria concentration was determined using a Petroff-Hausser counting chamber (Hausser Scientific®, 3900, Horsham, PA) and a phase contrast microscope at a magnification of 100 × .

### Gas production kinetics and statistical analyses

As previously detailed by Kholif et al. (2022), the kinetics of total gas, CO_2_, and CH_4_ production (mL/g DM) were estimated using the NLIN procedure of SAS (Online Version 9.4, SAS Inst., Inc., Cary, NC) according to France et al. (2000) model.

Data were analyzed using the GLM procedure of SAS (SAS Inst. Inc. Cary, NC, USA) in a complete randomized design using the model: *Y*_*ij*_ = *μ* + *R*_*i*_ + *ε*_*ij*_ where *Y*_*ij*_ is the observation, *μ* is the population mean, *R*_*i*_ is the replacement level effect, and *ε*_*ij*_ is the residual error. Data of each of the two runs of the same sample of the substrate were averaged prior to statistical analysis. Mean values of each individual run were used as the experimental unit. Linear and quadratic contrasts were used to examine responses to increasing replacement levels.

## Results

### Nutrient concentration

Replacing soybean meal with MOS gradually decreased the concentration of CP and increased NDF and ADF concentrations in rations (Table 1).

### Biogases production

**Fig. 1:**
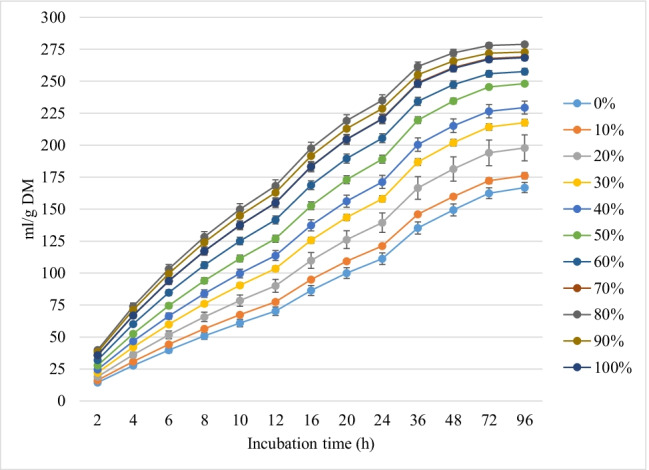
*In vitro* rumen gas production (mL/g incubated DM) of rations containing different levels of *M. oleifera* silage replacing soybean meal

**Fig. 2:**
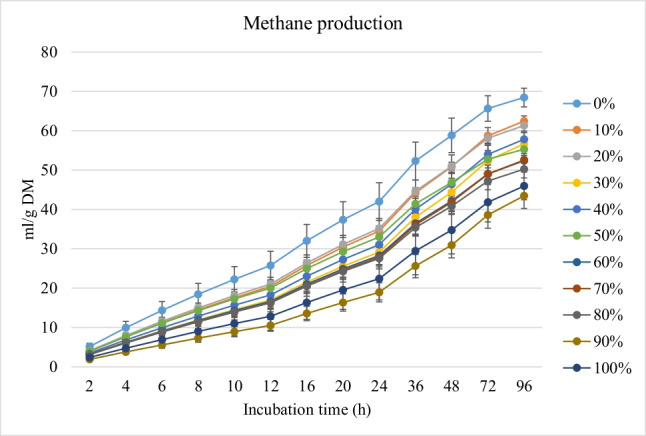
*In vitro* rumen methane production (mL/g incubated DM) of rations containing different levels of *M. oleifera* silage replacing soybean meal

**Fig. 3:**
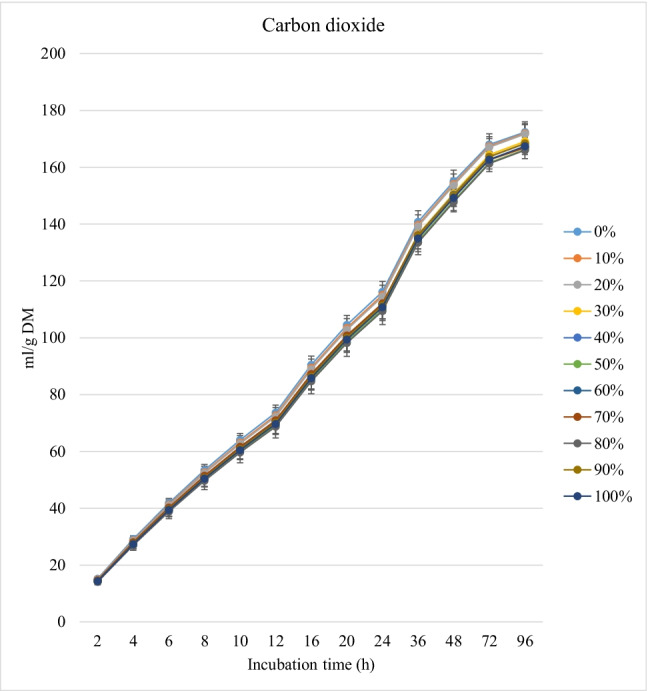
*In vitro* rumen carbon dioxide production (mL/g incubated DM) of rations containing different levels of *M. oleifera* silage replacing soybean meal

Figures 1, 2, and 3 show the *in vitro* rumen total gas, CH_4_, and CO_2_ production, respectively, from rations containing different levels of MOS replacing soybean meal at different incubation hours. Replacing soybean with MOS gradually increased (linear and quadratic effects, *P* < 0.001) the asymptotic GP and rate of GP while the lag time of GP was linearly (*P* < 0.01) increased (Table 2).

**Table 2:** *In vitro* rumen gas, methane (CH_4_), and carbon dioxide (CO_2_) kinetics of rations^1^ containing different levels of *M. oleifera* replacing soybean meal

Replacement^1^	Gas production^2^			CH_4_ production^3^				CO_2_ production ^4^	
	*b*	*c*	*Lag*	*b*	*c*	*Lag*	*b*	*c*	*Lag*
0% (Control)	169	0.044	1.38	71	0.039	1.41	175	0.045	1.44
10%	178	0.047	1.41	68	0.036	1.44	174	0.045	1.45
20%	200	0.050	1.44	65	0.033	1.46	174	0.044	1.36
30%	219	0.053	1.45	63	0.031	1.37	172	0.044	1.48
40%	230	0.056	1.46	61	0.028	1.49	171	0.044	1.51
50%	249	0.059	1.48	57	0.023	1.52	169	0.044	1.54
60%	258	0.066	1.51	55	0.020	1.56	169	0.045	1.55
70%	269	0.071	1.53	53	0.019	1.58	169	0.045	1.59
80%	279	0.077	1.55	51	0.018	1.62	169	0.043	1.61
90%	273	0.075	1.58	50	0.015	1.66	171	0.044	1.63
100%	269	0.071	1.65	47	0.013	1.69	170	0.044	1.66
SEM	2.3	0.0009	0.007	0.5	0.0006	0.034	1.4	0.0012	0.032
Linear	< 0.001	< 0.001	< 0.001	< 0.001	< 0.001	< 0.001	0.008	0.504	< 0.001
Quadratic	< 0.001	< 0.001	0.155	0.363	0.741	0.037	0.099	0.702	0.139

^1^* M. oleifera* silage replaced soybean meal at 0 to 100%, DM basis. SEM standard error of the mean

^2^*b*, the asymptotic gas production (mL/g DM); *c*, the rate of gas production (/h); *Lag*, the initial delay before gas production begins (h)

^3^*b*, the asymptotic CH_4_ production (mL/g DM); *c*, the rate of CH_4 _production (/h); *Lag*, the initial delay before CH_4_ production begins (h)

^4^*b*, the asymptotic CO_2_ (mL/g DM); *c*, the rate of CO_2 _production (/h); *Lag*, the initial delay before CO_2_ production begins (h)

Conversely, replacing soybean with MOS gradually decreased (linear effect, *P* < 0.001) the kinetic of biogases production (the asymptotic and the rates of CH_4_ and CO_2_ productions) and increased the lag time of CH_4_ and CO_2_ production, without affecting the rate of CO_2_ production (Table 2).

### Nutrient degradability and ruminal fermentation

Replacing soybean with MOS gradually increased (linear effect, *P* < 0.001) the digestibility of DM, NDF, and ADF, while it decreased the digestibility of CP (linear effect, *P* < 0.001) (Table 3).

**Table 3:** Degradability,* in vitro* rumen fermentation profile, and bacterial and protozoa counts of rations^1^ containing different levels of *M. oleifera* replacing soybean meal

Replacement^1^	Degradability^2^				Ruminal microorganisms^3^		Fermentation^4^		Volatile fatty acids^5^			
	DM	NDF	ADF	CP	Bacteria	Protozoa	pH	Ammonia-N	Total	Acetate	Propionate	Butyrate
0% (Control)	464	465	410	601	10.5	4.54	5.66	12	49	29	12	8.1
10%	528	498	422	600	10.9	3.79	5.69	12	50	30	12	8.9
20%	547	535	432	592	11.4	3.69	5.71	12	54	31	13	10.0
30%	559	554	443	591	11.8	3.49	5.71	11	56	32	14	9.4
40%	575	571	493	582	12.4	3.24	5.74	11	58	32	15	9.4
50%	575	582	511	587	12.9	3.12	5.75	11	60	33	14	10.0
60%	601	592	533	571	13.1	3.06	5.77	11	61	34	15	9.7
70%	640	611	549	575	13.8	2.90	5.86	11	61	34	15	9.7
80%	657	632	556	552	14.3	2.56	6.07	10	62	34	16	9.0
90%	617	623	560	547	13.6	2.29	6.18	10	63	35	15	9.1
100%	604	625	568	529	13.4	2.01	6.20	10	63	35	15	9.1
SEM	9.4	4.5	3.6	10.9	0.07	0.070	0.023	0.1	0.3	0.2	0.3	0.57
Linear	< 0.001	< 0.001	< 0.001	< 0.001	< 0.001	< 0.001	< 0.001	< 0.001	< 0.001	< 0.001	< 0.001	0.113
Quadratic	0.012	< 0.001	0.963	0.826	0.004	0.1437	< 0.001	0.718	< 0.001	0.063	0.003	0.221

^1^* M. oleifera* silage replaced soybean meal at 0 to 100%, DM basis. SEM standard error of the mean

^2^Degraded substrate (mg/g DM), DM, dry matter; *NDF*, neutral detergent fiber; *ADF*, acid detergent fiber

^3^Ruminal microorganisms (per mL incubation medium): Bacteria (total count × 10^8^), protozoa (total count × 10^5^)

^4^Ammonia-N (mg/g DM)

^5^Volatile fatty acids concentration (mmol/L)

A gradual increase in ruminal bacterial count (linear and quadratic effects, *P* < 0.01) and a gradual decrease in ruminal protozoal count (linear effect, *P* < 0.001) were observed when soybean meal was replaced with MOS (Table 3). Linear increases in fermentation pH (*P* < 0.001), and the concentrations of ruminal total VFA, acetate, and propionate, and linear decreases (*P* < 0.001) in the concentrations of ruminal ammonia-N were observed when MOS replaced soybean meal in the rations.

## Discussion

### Nutrient concentration

Replacing soybean meal with MOS gradually decreased dietary CP because MOS contained less protein (242 vs. 438 g/kg DM, respectively) and higher fiber than soybean meal (318 g NDF and 291 g ADF vs. 189 g NDF and 120 g ADF/kg DM, respectively). Chemical composition and nutrient concentration are among the major factors affecting the nutritive value of feeds and ruminal fermentation (Kholif et al. 2017).

### Biogases production

Ration containing MOS increased GP and rate of GP, indicating that increased fiber and decreased CP concentrations may be the main reasons for the increased gas production and lag time of GP. The simultaneous increase in total GP and lag time of GP indicates that most of the produced gas was lately produced as a result of late fiber fermentability and fiber is one of the main reasons for increased GP. Increased fiber and decreased CP contents in MOS diets may be another reason. Fermentation of protein gives a relatively small amount of gas compared to fermentation of carbohydrates (Makkar et al. 1995). This observation is not consistent with the observations of Kholif et al. (2017) who noted that increased fiber concentration in rations reduced total GP and increased lag time of GP. Moreover, this is inconsistent with the observations of Soliva et al. (2005) who found that replacing soybean meal and rapeseed meal with *M. oleifera* leaves decreased *in vitro* GP. The observed increased DM, NDF, and ADF digestibility and bacterial number with MOS rations may explain the increased GP as shown by Getachew et al. (2004) who confirmed a strong correlation between DM digestibility and total GP. The presence of small amounts of secondary metabolites in MOS, even at high inclusion levels, may be another reason. Generally, suitable (e.g., low and moderate) levels of plant secondary metabolites improve the activity of ruminal bacteria to degrade low and moderate concentrations of plant secondary metabolites (e.g., phenolic compounds and tannins) and utilize them as energy sources to digest feed and produce gases (Kholif and Olafadehan 2021).

Lowered production of CH_4_ and CO_2_ and rate of CH_4_ production with rations containing MOS is desirable from the environmental point of view. Moreover, increased lag time of CH_4_ and CO_2_ production with rations containing MOS was parallel with the result of increased GP. The secondary metabolites (e.g., tannins and phenolics) in MOS (Kholif and Olafadehan 2021) and variation in the chemical composition of the treatments (Soltan et al. 2021) may be responsible for these effects. In their experiments, Kholif et al. (2017) showed that chemical composition of incubated substrates affected *in vitro* production of CH_4_ and CO_2_ due to its effect on nutrient availability and microbial activity in the rumen. Secondary metabolites possess antimicrobial and protozoal properties which abate CH_4_ production (Kholif and Olafadehan 2021). Additionally, secondary metabolites affect ruminal cellulolytic bacteria (Patra and Saxena 2009) and reduce the formation of gases required for methanogenesis (i.e., CO_2_ and H_2_) (Goel and Makkar 2012). Bodas et al. (2012) noted that secondary metabolites in plants inhibit ruminal CH_4_-producing bacteria and decrease the concentrations of available H_2_ for methanogensis. Goel and Makkar (2012) observed a 50% reduction in the production of CH_4_ as a result of tannins and phenolic compound administration. The presence of α-linolenic acid in MOS (Ebeid et al. 2020b) may be another reason for decreased CH_4_ production (MacHmüller et al. 2000). Similar results were observed by Soliva et al. (2005) when they replaced soybean meal with *M. oleifera* leaves. The decrease in the protozoal count with MOS rations may be another reason for the lowered CH_4_ production (Bodas et al. 2012). Bodas et al. (2012) concluded that plant secondary metabolites decreased methanogenesis by approximately 8 to 14% under continuous culture conditions.

### Nutrient degradability and ruminal fermentation

Increased fiber and decreased CP in rations did not show any negative effect on nutrient digestibility. The increased digestibility of DM, NDF, and ADF with rations containing MOS may be related to the increased bacterial numbers. As previously mentioned, secondary metabolites and antioxidant properties present in *M. oleifera* can stimulate ruminal fibrolytic microbe activities and growth (Morgavi et al. 2000; Singla et al. 2021), resulting in faster degradation rate and extent of substrates (Kholif and Olafadehan 2021). Analysis of MOS for tannins and saponins showed that their concentrations (19 and 64 mg/g DM, respectively) were less than the critical levels which impair ruminal fermentation and feed digestibility (Frutos et al. 2004); Kholif and Olafadehan (2021). Ruminal microflora degrades and utilizes secondary metabolites at low and moderate levels and use them as energy sources without affecting ruminal fermentation (Frutos et al. 2004). Ebeid et al. (2020a) evaluated the degradability of nutrients in *M. oleifera* leaves and seeds and observed improved degradability of the leaves relative to the seeds.

The decrease in CP digestibility with replacing soybean meal by MOS may be a result of the presence of tannins and other phenolic compounds in the leaves of *M. oleifera* which can bind protein and decrease its ruminal degradation by ruminal microbes (Frutos et al. 2004; Kholif and Olafadehan 2021). Basha et al. (2014) observed a positive correlation between CP content and CP disappearance rate and a negative correlation between tannin concentration and CP disappearance. Additionally, the relatively high fiber content of MOS could bind N and decrease its availability to rumen microorganisms (Kendall et al. 1991; Ebeid et al. 2020a). This is a favorable effect from the nutritional point of view, as lower CP degradability indicates greater bypass protein that can be utilized in the duodenum (Barry and Manley 1986; Kumar et al. 1995). Ebeid et al. (2020a) evaluated the CP fermentation kinetics of *M. oleifera* leaves and seeds and observed a low effective degradability and high undegradable protein as well as a high intestinal CP digestibility.

It was expected that increasing MOS levels in the ration would decrease ruminal bacterial count, due to the antimicrobial effects of secondary metabolites, but this was not observed in the present experiment because rations containing MOS increased total ruminal bacterial counts. These results confirm our previous assertion that secondary metabolites in MOS are within acceptable ranges for ruminal bacterial activities. In their reviews and as previously noted, Frutos et al. (2004) and Kholif and Olafadehan (2021) reported high ability of ruminal microflora to utilize secondary metabolites as energy sources. The decreased ruminal protozoa with rations containing MOS partially possibly explains the reason for the high ruminal bacterial counts. Decreased protozoal population often decreases bacterial engulf, as ruminal protozoa are the main predators of bacteria in the rumen (Mathieu et al. 1996). The decrease in ruminal protozoal count with MOS may be related to the secondary metabolites in MOS. The presence of saponins and other plant secondary metabolites was reported to reduce methanogenic archaea and protozoa in rumen (Nowak et al. 2016; Kholif and Olafadehan 2021). Tannins were reported to have a strong defaunating effect, but the mode of action of this process is not clear (Bhatta et al. 2009). As previously noted, the low protozoal count resulted in decreased CH_4_ production.

The increased fermentation pH with MOS is a desirable effect because the activity of ruminal bacteria depends mainly on ruminal pH. In the present experiment, ruminal pH values were above the level recommended by Ryle and Ørskov (1990) for maximum ruminal bacterial activities, especially fiber digestion. Increased fiber contents of MOS rations must have been accompanied by increased salivation which invariably buffered the ruminal pH (Zebeli et al. 2012; Olafadehan et al. 2020). However, replacing soybean with MOS decreased the concentration of ruminal ammonia-N, with values being above the recommended level for maximum ruminal bacterial activities (Satter and Slyter 1974). The reduction of CP digestibility in MOS rations may be the main reason for the decreased ammonia-N concentration (Kholif et al. 2016). Additionally, the secondary metabolites in MOS, especially tannins, could reduce protein degradability in the rumen because tannins can bind dietary protein and protect it from ruminal degradation (Frutos et al. 2004). The decrease in total ruminal protozoal count cannot also be ignored as a reason for reduced ammonia-N concentration, because ruminal protozoa play an important role in protein degradation (Jouany 1996). Another possible reason for decreased ammonia-N is the inhibition of ammonia-producing bacteria by plant secondary metabolites (Newbold et al. 2004). The reduced ammonia-N concentration of MOS rations confirms the decreased rate and extent of proteolysis of CP of MOS compared to soybean meal. Earlier reports by Olafadehan and Adebayo (2016) attributed increased ammonia-N concentration of urea-ammoniated threshed sorghum tops vs. dried brewers’ grains to enhanced rate and degree of ruminal proteolysis of N from urea-ammoniated threshed sorghum tops.

The increased concentrations of total VFA, acetate, and propionate with MOS containing rations indicate improved ruminal fermentation, activity of ruminal bacteria, and feed digestion. In their review articles, Bodas et al. (2012) and Kholif and Olafadehan (2021) stated that plants containing secondary metabolites increased production of ruminal propionate and sometimes acetate as a result of improved digestion of structural and nonstructural carbohydrates.

## Conclusion

Soybean meal can be completely replaced with *M. oleifera* leave silage to increase gas production and decrease methane and carbon dioxide production, improve fiber digestibility, and decrease crude protein digestibility. Additionally, *M. oleifera* leave silage increased the concentrations of ruminal bacteria, total volatile fatty acids, acetate, and propionate and decreased the count of ruminal protozoa. Replacing soybean meal with *M. oleifera* leave silage up to 100% could be a valuable means of sustainable improvement of the environmental conditions through mitigation of methane and carbon dioxide emissions. Further studies are needed to establish the efficacy of replacement of soybean meal with *M. oleifera* leave silage in *in vivo* trials for production performance and greenhouse gas mitigation.

## Data Availability

The datasets used and/or analyzed during the current study are available from the corresponding author on reasonable request.
